# Whole gene sequencing identifies deep-intronic variants with potential functional impact in patients with hypertrophic cardiomyopathy

**DOI:** 10.1371/journal.pone.0182946

**Published:** 2017-08-10

**Authors:** Rita Mendes de Almeida, Joana Tavares, Sandra Martins, Teresa Carvalho, Francisco J. Enguita, Dulce Brito, Maria Carmo-Fonseca, Luís Rocha Lopes

**Affiliations:** 1 Instituto de Medicina Molecular, Faculdade de Medicina, Universidade de Lisboa, Lisbon, Portugal; 2 Departamento de Cardiologia, Hospital de Santa Maria, Centro Hospitalar Lisboa Norte, Centro Académico de Medicina de Lisboa, Lisbon, Portugal; 3 Centro Cardiovascular da Universidade de Lisboa, Lisbon, Portugal; 4 Institute of Cardiovascular Science, University College London, London, United Kingdom; Ohio State University Wexner Medical Center, UNITED STATES

## Abstract

**Background:**

High throughput sequencing technologies have revolutionized the identification of mutations responsible for genetic diseases such as hypertrophic cardiomyopathy (HCM). However, approximately 50% of individuals with a clinical diagnosis of HCM have no causal mutation identified. This may be due to the presence of pathogenic mutations located deep within the introns, which are not detected by conventional sequencing analysis restricted to exons and exon-intron boundaries.

**Objective:**

The aim of this study was to develop a whole-gene sequencing strategy to prioritize deep intronic variants that may play a role in HCM pathogenesis.

**Methods and results:**

The full genomic DNA sequence of 26 genes previously associated with HCM was analysed in 16 unrelated patients. We identified likely pathogenic deep intronic variants in *VCL*, *PRKAG2* and *TTN* genes. These variants, which are predicted to act through disruption of either splicing or transcription factor binding sites, are 3-fold more frequent in our cohort of probands than in normal European populations. Moreover, we found a patient that is compound heterozygous for a splice site mutation in *MYBPC3* and the deep intronic *VCL* variant. Analysis of family members revealed that carriers of the *MYBPC3* mutation alone do not manifest the disease, while family members that are compound heterozygous are clinically affected.

**Conclusion:**

This study provides a framework for scrutinizing variation along the complete intronic sequence of HCM-associated genes and prioritizing candidates for mechanistic and functional analysis. Our data suggest that deep intronic variation contributes to HCM phenotype.

## Introduction

Hypertrophic cardiomyopathy (HCM) is a genetic heart disease associated with sudden cardiac death and progressive heart failure. HCM is considered one of the most common genetic disorders, with an estimated prevalence of 1 in 500 people throughout the world [1]. Recognition of the disease is critical for providing treatment and prevention strategies as well as triggering clinical and genetic surveillance of family members [2],[3]. Mutation carriers may benefit from lifestyle and medical interventions that improve prognosis, whereas a negative genetic test can reassure individuals that are not at risk [3],[4].

Since a mutation in β-myosin heavy chain (*MYH7*) was first identified as the cause of HCM [5], other mutations affecting components of the sarcomere have been shown to have a pathogenic role in this disease [6]. In addition to *MYH7*, the most frequently mutated genes are cardiac myosin-binding protein C (*MYBPC3*), cardiac troponin T (*TNNT2*), cardiac troponin I (*TNNI3*), α-tropomyosin (*TPM1*), regulatory myosin light chain (*MYL2*), essential myosin light chain (*MYL3*) and cardiac actin (*ACTC1*) (**Table 1**). More rarely, mutations have been reported in other genes encoding proteins required for sarcomere structure and function, such as α-actinin 2 (*ACTN2*), muscle LIM protein (*CSRP3*) and calcium metabolism, such as phospholamban (*PLN*) and junctophilin 2 (*JPH2*) [7]. Additional sarcomere-related genes have been associated with HCM, although with less firmly established evidence for direct pathogenicity [8] (**Table 1**). These include α-myosin heavy chain (*MYH6*), telethonin (*TCAP*) [7], LIM domain binding 3 protein (*LDB3*) [9], myosin light chain kinase 2 (*MYLK2*) [10], myozenin 2 (*MYOZ2*) [11], nexilin (*NEXN*) [12], troponin C (*TNNC1*) [13], titin (*TTN*) [14], vinculin (*VCL*) [15], ankyrin repeat domain 1 (*ANKRD1*) [16] and caveolin 3 (*CAV3*) [17].

**Table 1:** Name of the genes analyzed, Ensembl accession number, and chromosomal position.

Sarcomere genes most frequently associated with HCM	Gene	Ensembl ID	Chromosome
β-Myosin heavy chain (thick filament)	*MYH7*	ENSG00000092054	14
Regulatory myosin light chain (thick filament)	*MYL2*	ENSG00000111245	12
Essential myosin light chain (thick filament)	*MYL3*	ENSG00000160808	3
Cardiac troponin T (thin filament)	*TNNT2*	ENSG00000118194	1
Cardiac troponin I (thin filament)	*TNNI3*	ENSG00000129991	19
α-Tropomyosin (thin filament)	*TPM1*	ENSG00000140416	15
α-Cardiac actin (thin filament)	*ACTC1*	ENSG00000159251	15
Cardiac myosin-binding protein C (intermediate filament)	*MYBPC3*	ENSG00000134571	11
**Sarcomere-related genes rarely associated with HCM**			
α-Actinin 2 (Z-disc)	*ACTN2*	ENSG00000077522	1
α-Myosin heavy chain (thick filament)	*MYH6*	ENSG00000197616	14
Muscle LIM protein (thin filament)	*CSRP3*	ENSG00000129170	11
Telethonin (Z-disc)	*TCAP*	ENSG00000173991	17
Phospholamban (calcium homeostasis)	*PLN*	ENSG00000198523	6
Junctophilin 2 (calcium homeostasis)	*JPH2*	ENSG00000149596	20
Ankyrin Repeat Domain 1 (titin-associated)	*ANKRD1*	ENSG00000148677	10
Caveolin 3 (membrane scaffold)	*CAV3*	ENSG00000182533	3
LIM Domain Binding 3 (Z-disc)	*LDB3*	ENSG00000122367	10
Myosin light chain kinase 2 (regulation of muscle contraction)	*MYLK2*	ENSG00000101306	20
Myozenin 2 (Z-disc)	*MYOZ2*	ENSG00000172399	4
Nexilin F-actin binding protein (thin filament)	*NEXN*	ENSG00000162614	1
Troponin C, slow (thin filament)	*TNNC1*	ENSG00000114854	3
Titin (sarcomere scaffold)	*TTN*	ENSG00000155657	2
Vinculin (thin filament)	*VCL*	ENSG00000035403	10
**Non-sarcomere genes associated with HCM phenocopies**			
Protein kinase, AMP-activated, γ 2 subunit (Wolff-Parkinson-White syndrome)	*PRKAG2*	ENSG00000106617	7
Lysosomal-associated membrane protein 2 (Danon disease)	*LAMP2*	ENSG00000005893	X
Galactosidade, α (Fabry disease)	*GLA*	ENSG00000102393	X

Some rare inherited diseases may mimic the phenotypic and clinical features of sarcomere HCM, as defined by the presence of unexplained left ventricular hypertrophy. These conditions are referred to as HCM phenocopies and represent distinct disease entities with respect to inheritance, pathophysiology, natural history, extra-cardiac features, and management [3], [18]. These disorders are not caused by sarcomeric mutations. The most prominent HCM phenocopies in adults include [3] Fabry disease, caused by mutations in the galactosidase-α gene (*GLA*); Danon disease, a lysosomal storage disease caused by mutations in the lysosomal-associated membrane protein 2 gene (*LAMP2*); and LVH associated with Wolff-Parkinson-White syndrome, caused by mutations in the regulatory subunit of adenosine monophosphate-activated protein kinase gene (*PRKAG2*) (**Table 1**).

In recent years, widespread availability of genetic testing has proved crucial not only to identify the sarcomeric mutations that cause HCM but also to distinguish disorders that can mimic HCM [2, 4]. However, despite the revolutionary increase in genetic testing capability introduced by next-generation sequencing [19], approximately 50% of individuals with a clinical diagnosis of HCM have no causal mutation identified [4, 20],[21].

One possibility to explain why many individuals fail to be genetically diagnosed is the presence of deep-intronic mutations undetected by current clinical genetic testing approaches, which provide information restricted to the exons and exon-intron boundaries. Indeed, recent Genome Wide Association Studies have identified many single nucleotide variants located deep within introns with significant association to diseases [22, 23]. To date, over 180 deep intronic pathogenic variants located at least 100 bp from the nearest canonical splice site have been reported across 77 different disease genes [24]. Most frequently, these mutations lead to pseudo-exon inclusion due to activation of non-canonical splice sites or changes in splicing regulatory elements [25, 26]. The more common mechanism involves a mutation that creates a novel donor splice site and activates a pre-existing non-canonical acceptor splice site, whereas more rarely the mutation creates a novel acceptor splice site. Alternatively, inclusion of cryptic exons can be induced by mutations that either create an enhancer sequence element or inactivate a repressive sequence [24]. For example, a deep intronic mutation (c.639+919G>A) in the galactosidase alpha (*GLA*) gene, responsible for Fabry disease, disrupts an hnRNP A1 and hnRNP A2/B1-binding splicing silencer motif, thus allowing binding of U1 snRNP to an overlapping cryptic 5'ss that results in pseudo-exon inclusion [27]. The appearance of a pseudo-exon generally disrupts the reading frame introducing a premature termination codon that targets the mutant mRNA for degradation by nonsense mediated decay (NMD) [28], making mutations that result in abnormal splicing functionally equivalent to null or hypomorphic alleles. In other cases of genetic diseases caused by deep intronic variation, the mutation disrupts transcription regulatory motifs leading to decreased expression of the affected gene [24].

In this study, we used targeted high throughput sequencing and computational approaches to identify deep intronic variants that may contribute to HCM phenotype.

## Methods

### Patients

The study population comprised 16 unrelated consecutively evaluated patients (8 males, 8 females) referred to the Cardiology Department at University Hospital Santa Maria. For all probands, the personal and family history, physical examination, ECG and echocardiography were consistent with a diagnosis of HCM according to international criteria [3]. Patients were genetically tested at a mean age of 49 years. In addition, family members of two selected probands were clinically and genetically tested. Before blood collection, all patients and relatives provided written informed consent for DNA analysis and received genetic counselling in accordance with guidelines [3]. DNA samples used in this study were residual after conventional diagnostic screening by targeted exome and Sanger sequencing. The project was approved by the Lisbon Academic Medical Center Ethics Committee.

### Targeted gene enrichment and sequencing

Blood samples (5–8 mL) were collected into EDTA tubes at routine clinic visits, and DNA was isolated from peripheral blood lymphocytes using standard methods. The study was designed to screen the full genomic DNA sequence of 26 genes indicated in **Table 1**. These genes are included in many commercially available testing panels. A capture library was designed using SureSelect (Agilent) and target regions were sequenced (paired-end) on an Illumina HiSeq platform with 30–97 base read length. Highly repetitive sequences were excluded. Sample preparation was carried out as recommended by the manufacturer. Relatives were genotyped for selected variants by Sanger sequencing.

### Bioinformatic data analysis

**Fig 1:**
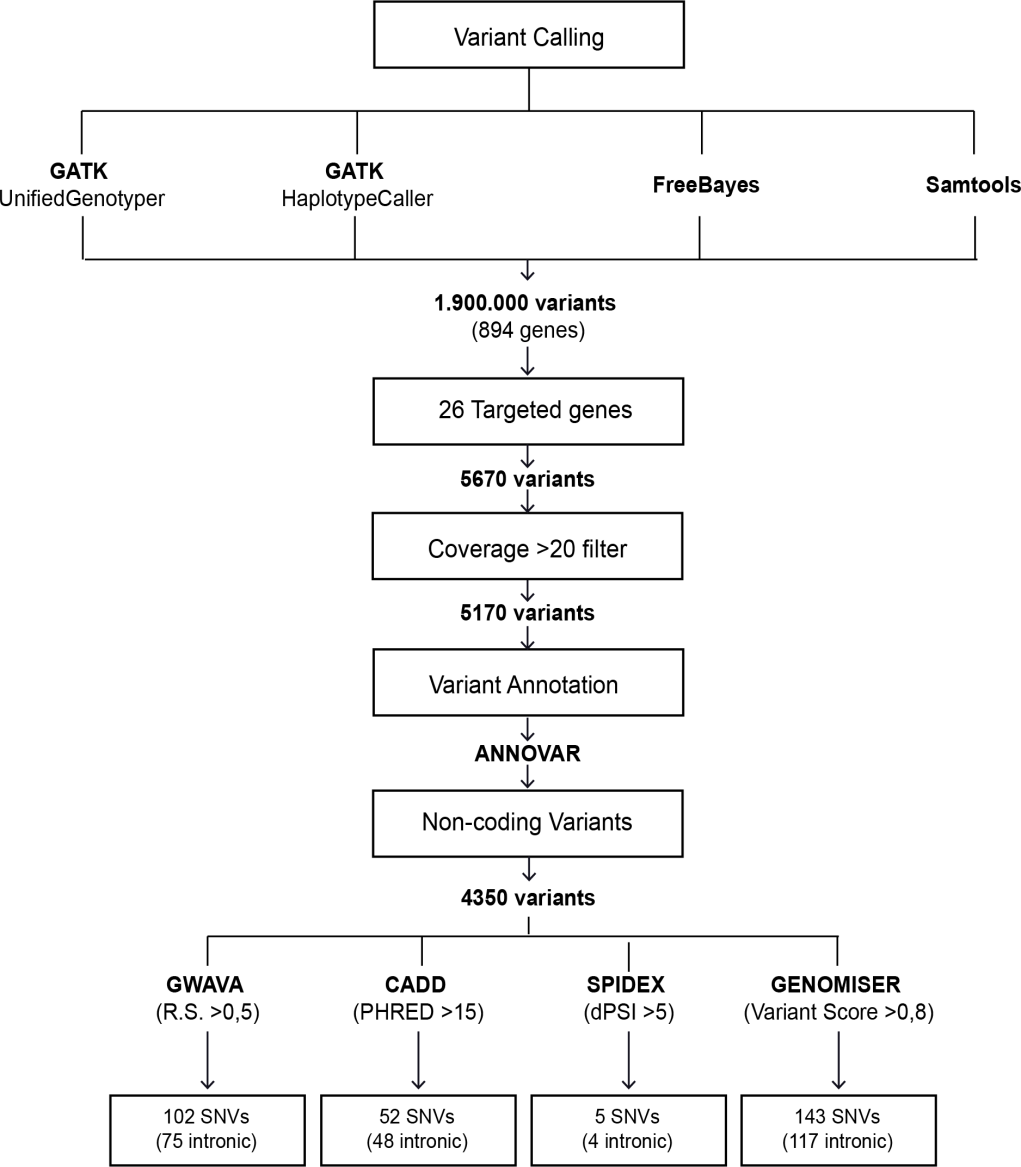
Flowchart of noncoding data analysis. Variants that were independently selected by at least two tools and presented a read depth of 20 or more in the 26 targeted genes were annotated with ANNOVAR. *In silico* predictions were carried out for noncoding variants that were not classified as either benign/likely benign or pathogenic/likely pathogenic in NCBI ClinVar. All variants with scores above the indicated threshold were single nucleotide substitutions (SNVs). GWAVA: Genome-Wide Annotation of Variants. CADD: Combined Annotation Dependent Depletion. SPIDEX: Splicing Index. R.S.: Region score. PHRED: phred quality score. dPSI: percent of spliced in.

Raw sequencing paired-end reads (in.*fastq* format) were aligned using BWA software (version 0.7.12) [29] on the human reference genome (GRCh37) using quality score calibration and Illumina adapter trimming. Following the exclusion of duplicate reads using Picard MarkDuplicates tool (version 1.96) (http://broadinstitute.github.io/picard/), regions around insertion-deletions (indels) were realigned and each base quality score was recalibrated. For variant calling, we used four distinct tools: GATK-UnifiedGenotyper (version 3.4–46) [30] and SAMtools mpileup (version 1.2) [31], which use alignment-based approaches, and GATK-HaplotypeCaller (version 3.4–46) [30] and FreeBayes (version 0.9.21.26) [32], which use haplotype-based approaches. By comparing the performance of each tool against a standard reference (NA12878, published by Genome in a Bottle consortium [33], we observed a concordance of ~85% (**S1 Fig**). To take advantage of the strengths of the different tools, we selected variants that were independently called by at least two of them. This strategy showed a better sensitivity (~97%) and precision (~98%) compared to analysis using a single tool (**S1 Fig**). Variants that were independently selected by at least two tools, and presented a read depth of 20 or more in the targeted genes, were annotated with ANNOVAR [34] (**Fig 1**).

For analysis of the clinical impact of coding variants we used the NCBI ClinVar database (http://www.ncbi.nlm.nih.gov/clinvar/) [35] and classified the variants according to the American College of Medical Genetics and Genomics (ACMG) guidelines [36]. Prediction of pathogenicity was performed with SIFT [37], PolyPhen2 HVAR [38], Human Splicing Finder (version 3.0) [39], Mutation taster [40], UMD-predictor [41], PROVEAN [42] and FATHMM [43]. Prioritization of noncoding variants was achieved using GWAVA (version 1.0) [44], CADD (version 1.3) [45], SPIDEX [22] and Genomiser [46]. To determine whether a variant may disrupt splicing motifs we used Human Splicing Finder (version 3.0), a tool that predicts potential splice sites, branch points and enhancer/silencer splicing motifs [39]; RegRNA (version 2.0), which searches for enhancer/silencer splicing motifs [47]; and Regulatory Genomics: Branch point analyser, that predicts the presence of branch points and respective polypyrimidine tracts [48].

As deep intronic mutations may result in altered gene expression through either cryptic splicing or disruption of transcription regulatory motifs [24], we investigated whether the identified intronic variants may disrupt transcription factor binding sites (TFBS). We used available tracks in the UCSC genome browser [49–51] (Transcription Factor ChIP-seq Uniform Peaks from ENCODE/Analysis and HMR Conserved Transcription Factor Binding Sites), focusing on TFBS predicted to be targets for transcription factors that have been implicated in pathways related to cardiac regulation, development or pathophysiology.

Variant frequency was determined using the allele frequency estimates from the 1000 genomes project [52] and gnomAD [53] databases (accessed on June 2017).

Finally, we searched for the potential association of the candidate deep intronic variants with cardiac diseases identified through GWAS (https://www.genome.gov/gwastudies/index.cfm?gene=ESRRG and http://www.ebi.ac.uk/gwas/).

## Results

### Quality of sequencing data

Analysis of sequencing data yielded an average of 96.64% confidently mapped reads per gene. For 69% of the targeted genes the average read depth was above 200, and for the remaining genes the average read depth ranged between 130 and 200 (**S2A Fig**). The average read depth was slightly lower over noncoding regions (**S2B Fig**). The average percentage of covered base pairs was higher than 90 for 85% of the genes, and the lowest coverage was 76% for both coding and noncoding regions (**S2 Fig**). Following alignment to the reference genome (GRCh37) and variant calling, we removed variants that were off-target, or had an average read depth below 20 (**Fig 1**). Single nucleotide substitutions and insertions or deletions of a few bases were identified and considered for further analysis.

### Spectrum of exonic and splice site variants

Previously described disease-causing variants in the *MYBPC3* gene were detected in 3 patients (**Table 2**). Rare variants classified in the NCBI ClinVar database and according to the ACMG guidelines as of uncertain significance were additionally detected in the *TNNT2*, *MYBPC3*, *TTN*, *TPM1* and *MYH6* genes; all these scored as likely pathogenic according to multiple *in silico* prediction tools (**Table 2**). Noteworthy, one of the patients harboured, in addition to *MYH6* variant rs140596256, a novel variant in the *GLA* gene that is not listed in online databases but is predicted to be pathogenic by multiple prediction tools (**Table 2**).

**Table 2:** Putative HCM-causing variants located in exons and exon-intron boundaries. VUS, variant of uncertain significance. ACMG, American College of Medical Genetics and Genomics.

Patient #	Exonic and splice-site variants	ClinVar/Reference	ACMG classification	*In silico* predictions
1	*TNNT2*: c.198G>C (p.Lys76Asn) Het (rs727504869)	ClinVar—VUS	VUS	• UMD-predictor: probably pathogenic • SIFT: deleterious • PROVEAN: damaging • PolyPhen2 HVAR: probably damaging • FATHMM: damaging • Mutation Taster: disease causing
2	*MYBPC3*: c.1224-19G>A Het (rs587776699)	ClinVar—Conflicting interpretations	VUS	• Mutation Taster: disease causing • Human Splicing Finder: activation of intronic cryptic acceptor site
5	*TTN*: c.57478C>G (p.Leu19160Val) Het (rs781121273)	ClinVar- VUS	VUS	• UMD-predictor: probably pathogenic • SIFT: deleterious • FATHMM: damaging • Mutation Taster: disease causing
6	*MYBPC3*: c.1227-13G>A Het (rs397515893)	[58]	Pathogenic (IC)	
7	*TPM1*: c.62G>T (p.Arg21Leu) Het (rs730881151)	ClinVar—Conflicting interpretations	VUS	• UMD-predictor: pathogenic • SIFT: deleterious • FATHMM: damaging • Mutation Taster: disease causing
8	*MYBPC3*: c.2827C>T (p.Arg943*) Het (rs387907267)	[60]	Pathogenic (ID)	
12	*TPM1*: c.841A>G (p.Met281Val) Het (rs397516394)	ClinVar—VUS	VUS	• UMD-predictor: pathogenic • SIFT: deleterious • PROVEAN: damaging • FATHMM: damaging
14	*MYH6*: c.292G>A (p.Glu98Lys) Het (rs140596256)	ClinVar—VUS	VUS	• UMD-predictor: pathogenic • SIFT: deleterious • PROVEAN: damaging • PolyPhen2 HVAR: probably damaging • FATHMM: damaging
	*GLA*: c.187T>A (p.Cys63Ser) Het	[62]	VUS	• UMD-predictor: pathogenic • PolyPhen2 HVAR: probably damaging • FATHMM: damaging Mutation Taster: disease causing
15	*MYBPC3*: c.1484G>A (p.Arg495Gln) Het (rs200411226)	[61]	Likely pathogenic (III)	
16	*MYH6*: c.292G>A (p.Glu98Lys) Het (rs140596256)	ClinVar—VUS	VUS	• UMD-predictor: pathogenic • SIFT: deleterious • PROVEAN: damaging • PolyPhen2 HVAR: probably damaging • FATHMM: damaging

### Assessment of deep intronic variants

The noncoding variants were prioritized using GWAVA [44], CADD [45], SPIDEX [22], and Genomiser [46]. The genome-wide annotation of variants (GWAVA) is a computational approach that integrates a wide range of available genomic and epigenomic annotations to predict the functional impact of variants. GWAVA results are in the range 0–1, with higher values indicating variants predicted as more likely to be functional. Variants with a GWAVA score above 0.5 were classified as functional, as in previous studies [44]. The Combined Annotation-Dependent Depletion (CADD) method provides a metric (C score) for deleteriousness, a property that strongly correlates with functionality and pathogenicity [45]. Variants at the top 10% of deleteriousness are assigned a C score of 10, whereas variants at the top 1% are assigned a C score of 20. Variants with C score greater than 15 were selected, as previously described [54]. SPIDEX is a computational model that uses the Percentage of Spliced-In (PSI) metric to evaluate whether a certain splicing isoform is more enriched under the presence/absence of a given variant. SPIDEX scores higher than 5 predict that the variant affects RNA splicing [22]. The Genomiser framework combines a machine learning method and an integrative algorithm for ranking noncoding variants in whole-genome sequence data [46]. Genomiser results are in the range 0–1, with values higher than 0.6–0.9 indicating variants more likely to be pathogenic [46].

**Fig 2:**
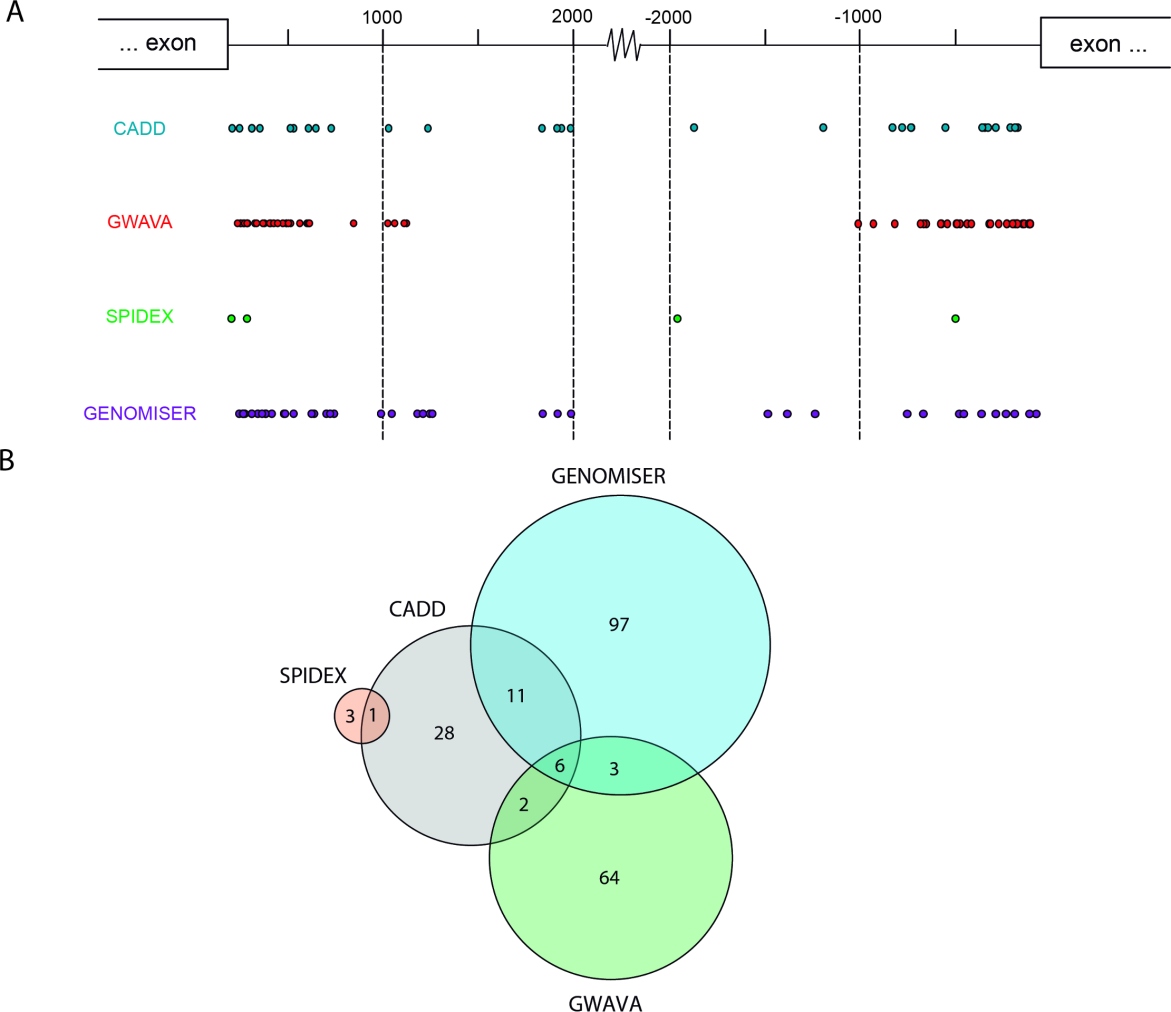
Assessment of intronic variants. **A)** Schematic diagram depicting the position of intronic variants prioritized by each prediction tool. **B)** Venn diagram illustrating intronic variants that are simultaneously prioritized by multiple prediction tools.

We found that all noncoding variants with higher scores for the different prediction metrics correspond to single nucleotide substitutions, the vast majority of which are located within introns (**Fig 1**). The position of each variant relative to the nearest canonical splice site ranged between 20 and 2000 nucleotides (**Fig 2A**). A comparison of variants prioritized as likely pathogenic by the different tools resulted in the identification of 6 variants that scored with high values using GWAVA, CADD and Genomiser metrics (**Fig 2B**). These include two variants in *VCL*, two variants in *TTN*, one variant in *ACTC1* and one variant in *PRKAG2* (**Table 3**). Analysis of allele frequency data available for European populations in the 1000 Genomes project [52] and gnomAD [53] databases reveals that two of these variants are more frequent in the patient population than in healthy individuals (**Table 3**). Namely, the *VCL* variant c.499+367T>C (rs113195070) was detected in 6 probands, corresponding to an allele frequency of 19% in the patient population. This contrasts with a frequency of 6–7% in control populations. Similarly, the *PRKAG2* variant c.1234-317T>G was present with an allele frequency of 3% in the patient population contrasting with a frequency of 0.1–0.3% in healthy individuals. Such specific enrichment of certain deep intronic variants in the patient population suggests that these may be contributing to the disease phenotype.

**Table 3:** Prioritized intronic variants. For each variant, minor allele frequency (MAF) was determined using European populations in the 1000 genomes project database (52) and gnomAD database (53). CADD Phred score (45); GWAVA Region score (44); Genomiser Variant score (46); SPIDEX dPSI score (22). HGVS, Human Genome Variation Society.

Tools	Gene symbol	dbSNP ID	HGVS	CADD score	GWAVA score	GENOMISER score	SPIDEX score	MAF 1000G	MAF gnomAD	Frequency in probands
CADD, GWAVA, GENOMISER	*VCL*	rs77884406	c.169-2410A>C	17.55	0.59	0.910891	NA	0.019	0.0273	0.03125
	*VCL*	rs113195070	c.499+367T>C	16.93	0.6	0.963367	NA	0.06	0.0671	0.1875
	*ACTC1*	rs28595759	c.129+472T>C	21.9	0.53	0.9792082	NA	0.07	0.0604	0.03125
	*TTN*	rs2243452	c.32929+72T>C	22.2	0.53	0.939604	0.6211	0.029	0.0247	0.03125
	*TTN*	rs2253324	c.10361-138C>T	18.03	0.51	0.825743	3.3164	0.048	0.0441	0.03125
	*PRKAG2*	rs141541040	c.1234-317T>G	15.20	0.58	0.872277	NA	0.003	0.0010	0.03125
CADD, GENOMISER	*VCL*	rs7079796	c.168+1165C>T	15.74	0.35	0.812872	NA	0.2	0.2191	0.15625
	*VCL*	-	c.169-7572C>T	15.88	NA	0.905941	NA	.	.	0.03125
	*VCL*	-	c.239+4299C>A	18.78	NA	0.983169	NA	.	0.0001	0.0625
	*LDB3*	rs12570315	c.93+1827G>A	16.75	0.23	0.858416	NA	0.3	0.3436	0.25
	*LDB3*	-	c.548+1914C>T	21.4	NA	0.970297	NA	.	.	0.03125
	*LDB3*	rs779483568	c.548+1993C>T	17.35	NA	0.990099	NA	.	0.0001	0.03125
	*MYL2*	rs2040571	c.3+604C>T	15.58	0.36	0.89703	NA	0.086	0.0888	0.03125
	*PRKAG2*	rs62478182	c.467-44847T>G	17.95	0.26	0.880198	NA	0.34	0.3721	0.25
	*PRKAG2*	rs114394151	c.115-30242C>T	18.38	0.49	0.925743	NA	.	0	0.0625
	*LAMP2*	rs5956217	c.1094-2886A>G	15.93	0.42	0.881188	NA	0.004	0.0009	0.03125
	*LAMP2*	rs42887	c.1094-2924C>T	20.4	0.43	0.929703	NA	0.11	0.1711	0.125
CADD, GWAVA	*TTN*	rs12693162	c.37112-700G>A	18.66	0.5	0.190099	NA	0.22	0.2261	0.21875
	*LAMP2*	rs141348126	c.1094-140A>G	15.68	0.5	0.545545	NA	.	0	0.03125
GWAVA, GENOMISER	*VCL*	rs2131959	c.2132-437G>C	10.13	0.57	0.89604	NA	0.75	0.7445	0.84375
	*ANKRD1*	rs10509614	c.207+239G>T	13.87	0.52	0.838614	0.7561	0.04	0.0314	0.03125
	*TTN*	rs80259697	c.10360+317T>C	13.04	0.51	0.821782	NA	.	6.68e-05	0.03125
CADD, SPIDEX	*TTN*	rs142156368	c.31484-286G>T	15.63	0.38	0.425	6.092	0.0089	0.0047	0.0625
SPIDEX	*TTN*	rs2562845	c.32593+111A>G	3.232	0.29	0	9.1111	0.21	0.2015	0.15625
	*TTN*	rs72650063	c.32077+31C>G	0.713	0.41	0.019802	5.8489	0.021	0.0218	0.0625
	*TTN*	rs2742353	c.31484+1715A>C	10.79	0.26	0.556436	6.2975	0.029	0.0247	0.03125

**Fig 3:**
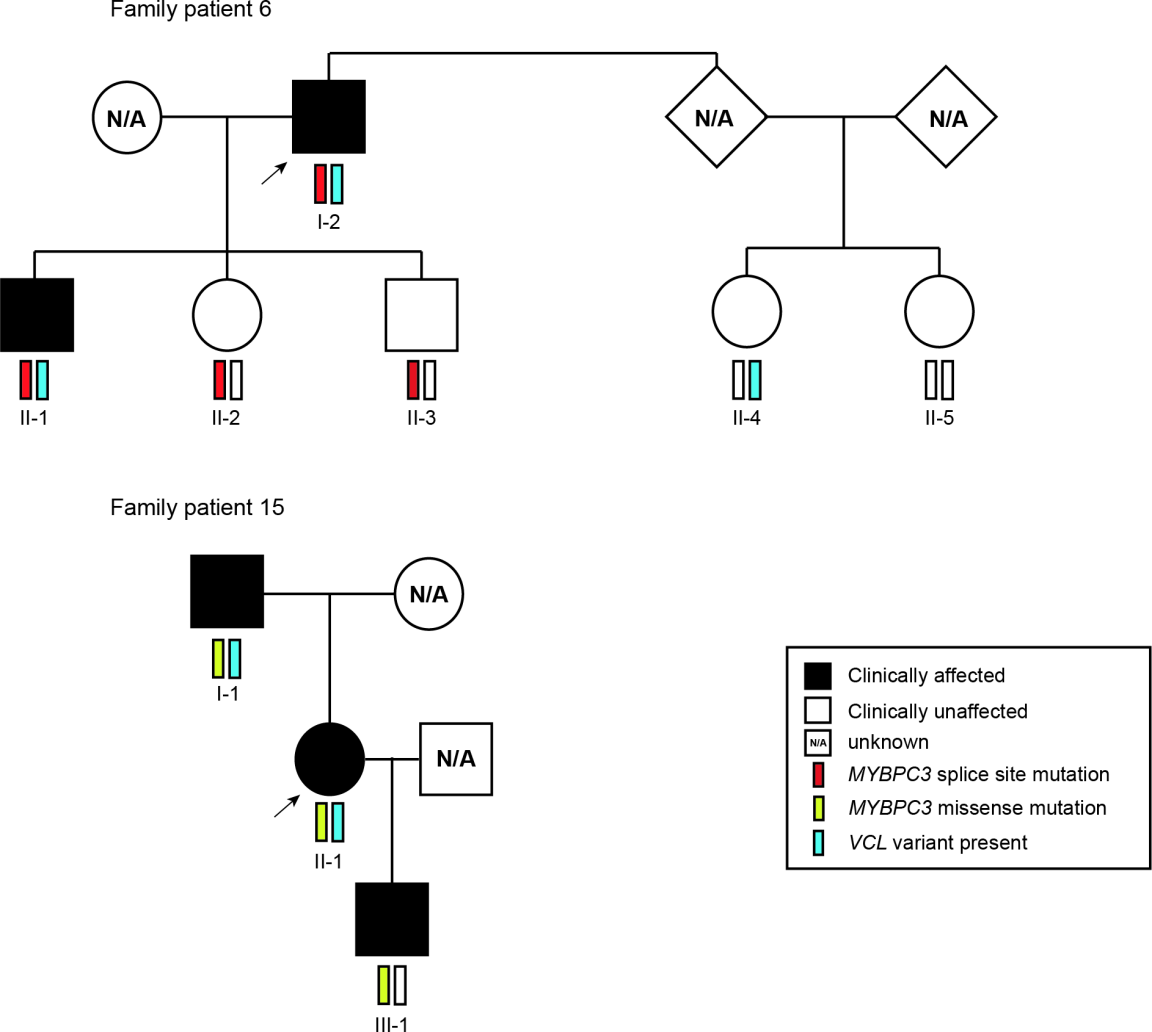
Family pedigrees. The *MYBPC3* splice site (c.1227-13G>A Het) and missense (p.Arg495Gln) mutations, and the *VCL* variant (c.499+367T>C) identified in probands (arrows) were studied in family members, and their clinical status was ascertained. Circles denote females, squares males, solid symbols clinically affected individuals, open symbols clinically unaffected individuals, and NA unknown clinical status.

We focused on the *VCL* variant c.499+367T>C. We found that two of the probands were compound heterozygous for this variant and a *MYBPC3* mutation previously described as disease-causing. Genotyping of family members of proband #6 showed that the dual presence of the *MYBPC3* splice site mutation (c.1227-13G>A) and the *VCL* variant is associated with the manifestation of the phenotype in the proband (I-2) and his son (II-1), both diagnosed in their 40s (**Fig 3**). The other children of the proband (II-2 and II-3), while carrying the *MYBPC3* mutation, did not develop signs of cardiomyopathy when assessed at a similar age. This suggests a possible modifier effect of the *VCL* variant, since the presence of the *MYBPC3* mutation alone is not sufficient for the phenotype to be manifested. Analysis of this family further suggested that the *VCL* variant on its own is not sufficient to cause disease. Genotyping of family members of proband #15 (**Fig 3**) indicated that presence of the *MYBPC3* missense mutation (p.Arg495Gln) in the absence of the *VCL* variant appears sufficient to cause the phenotype, even at a pediatric age (III-1). Clinical characteristics for the two families are detailed in **Table 4**.

**Table 4:** Clinical, electrocardiographic and echocardiographic data for the proband and relatives of families 6 and 15 (pedigrees are illustrated in Fig 3).

	Status	Age at clinical diagnosis or genetic testing (y)	Symptoms** (Yes/No)	Abnormal ECG (Yes/no)	LVH (Yes/No)	Maximal WT (mm)	Type of LVH	OB	LA (mm)	LVDD (mm)	LVSD (mm	FS (%)	LVEF	Vs´ (lateral; cm/s)	E/e’ (lateral; cm/s)
Family 6															
I-2	G+/Ph+	49	Yes	Yes	Yes	30	ASH	Yes	48	40	20	50	82	4	6.85
II-1	G+/Ph+	44*	No	Yes	Yes	16	ASH	No	32	44	23	57	87	9	5.9
II-2	G+/Ph-	42*	No	No	No	11	-	-	33	44	23.6	46	76	14	4.5
II-3	G+/Ph-	40*	No	No	No	12	-	-	41	48	25.6	47	78	13	6.18
II-4	G-/Ph-	43	-	-	-	9	-	-	28	43	21.6	52	83	10	6.6
II-5	G-/Ph-	40	-	-	-	11	-	-	32	41	22	46	78	12	5.33
Family 15															
II-1	G+/Ph+	20	Yes	Yes	Yes	19.8	ASH	No	41	39	21.35	44	66	10	7.38
I-1	G+/Ph+	21	Yes	Yes	Yes	15	ASH	No	46	42	22	48	62	9	6.9
III-1	G+/Ph+	3*	No	Yes	Yes	8.8 £	ASH	No	22.34	32	19.7	40	72	7	6.63

G+ = carrier of the causative mutation in the *MYBPC3* gene; G- = not carrier of the causative mutation; Ph+ = positive phenotype; Ph- = negative phenotype; Hypertrophic cardiomyopathy (HCM)

*diagnosis by familial screening (not symptoms)

**symptoms related to HCM

ASH = asymmetrical septal hypertrophy; LVH = left ventricular hypertrophy; & maximal wall thickness (WT) in any left ventricular segment; LA = left atrial dimension (M-mode echocardiography); LVDD = left ventricular diastolic diameter; LVSD = left ventricular systolic dimension; FS = fractional shortening of the left ventricle; LVEF = left ventricular ejection fraction (Simpson method); OB = presence of left ventricular obstruction at rest (left ventricular outflow gradient ≥ 30 mmHg on Doppler evaluation); £ - left ventricular hypertrophy considering pediatric criteria for HCM; Vs = Velocity of the mitral annulus (lateral) by Tissue Doppler imaging (TDI); E/e = ratio of early diastolic velocity of mitral inflow to early diastolic velocity of the mitral annulus (lateral) by TDI.

**Fig 4:**
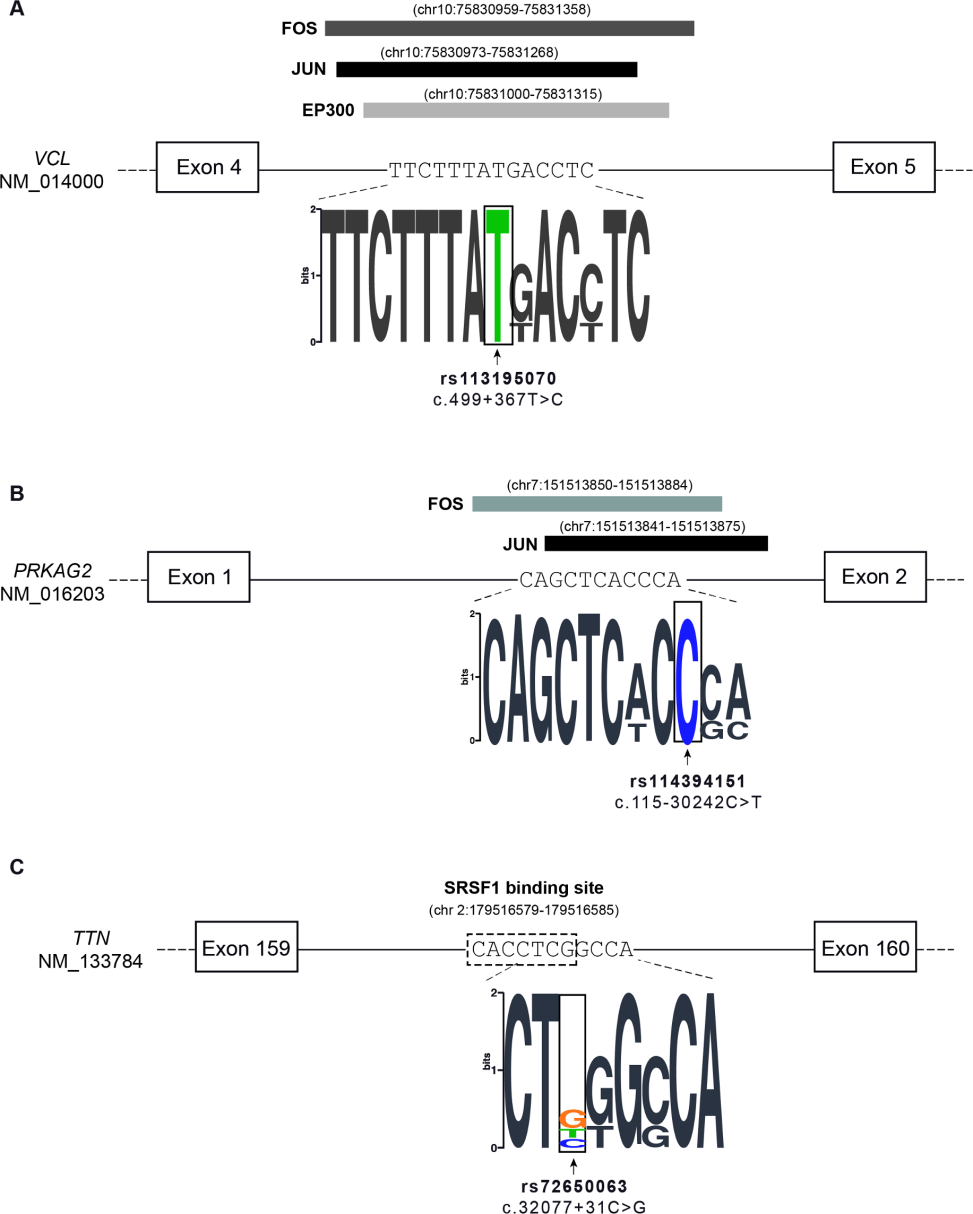
Variants located at binding sites for transcription and splicing factors. **A)** The *VCL* variant c.499+367T>C (rs113195070) is located at a binding site for transcription factors FOS, JUN and EP300. **B)** The *PRKAG2* variant c.115-30242C>T (rs114394151) is located at a binding site for transcription factors FOS and JUN. **C)** The *TTN* variant c.32077+31C>G (rs72650063) is predicted to disrupt the binding site of splicing factor SRSF1.

We found that, based on Chip-seq experiments [49, 50], the deep intronic *VCL* variant enriched in the patient population (rs113195070) localizes in a region associated with FOS, JUN and EP300 (**Fig 4A**). A deep intronic variant in the *PRKAG2* gene (rs114394151) prioritized by CADD and Genomiser is enriched in the patient population (**Table 3**) and localizes in a region associated with FOS and JUN (**Fig 4B**). FOS and JUN transcription factors are thought to be among the first set of genes to be expressed in the context of pathological cardiac hypertrophy [55], and EP300 has been associated with cardiomyocyte enlargement [56].

Finally, using the SPIDEX tool we identified a variant in the *TTN* gene (rs72650063) that occurs with a frequency of 2% in control European populations and is present in two probands, corresponding to an allele frequency of 6% in the patient population (**Table 3**). This variant is predicted by Human Splicing Finder to disrupt binding of splicing factor SRSF1 (**Fig 4C**) [57]. Another variant in the *TTN* gene (rs142156368) appears highly enriched in our cohort relative to the general population (**Table 3**).

No potential association of the candidate deep intronic variants with cardiac diseases identified through GWAS was found.

## Discussion

Motivated by the clinical heterogeneity of HCM and the lack of a conclusive genetic diagnosis in approximately 50% of the patients [4, 20],[21], we hypothesized that genetic variation within deep intronic regions of sarcomere and sarcomere-related genes contributes to the disease mechanism. Using a targeted high-throughput sequencing strategy, we did a comprehensive screening of 26 genes in a cohort of 16 unrelated HCM patients using recently developed computational models to assess variants.

We identified 3 probands carrying previously described disease-causing variants in the *MYBPC3* gene [58–61], in agreement with the finding that mutations in this gene account for the great majority (30–40%) of identified genetic causes of HCM [3],[7]. We further identified additional probands harbouring rare coding variants of uncertain significance, that are likely pathogenic as assessed by multiple prediction tools, in *TNNT2*, *MYBPC3*, *TPM1*, *TTN* and *MYH6* genes. In one patient we identified a novel variant in the *GLA* gene, associated with Fabry disease [62].

In agreement with previous studies on larger HCM patient cohorts [61, 63], we identified missense and stop-codon mutations, as well as splice site mutations (**Table 2**). While missense mutations may code for a pathogenic protein that would cause HCM by a gain-of-function mechanism, stop-codon and splice site mutations are more likely to act through a loss-of-function mechanism due to reduced levels of the normal protein. Demonstrating that particular mutations act through a dominant gain-of-function mechanism and others cause loss-of-function would be critical to understand phenotype-genotype correlations.

In this regard, we analysed the family members of two probands with either a missense or a splice site mutation in *MYBPC3* (**Fig 3**) and found that all carriers of the missense mutation (family #15) were clinically affected, as expected for a dominant gain-of-function mechanism. In contrast, in the other family, two individuals are carriers of the splice site mutation (family #6) and do not manifest the disease, consistent with a loss-of-function model. Possibly, the *MYBPC3* splice site mutation present in family members of proband #6 results in mis-splicing by either decreasing the specificity or fidelity of splice site selection or activating cryptic splice sites that are normally not used. Abnormal splicing often results in a frameshift and consequent introduction of premature termination codons (PTCs), which trigger degradation of the mRNA by nonsense-mediated decay [28]. Thus, this mutation can be functionally equivalent to a null or hypomorphic allele associated with loss-of-function of the protein. In contrast, the *MYBPC3* missense mutation present in family members of proband #15 presumably leads to an abnormal protein containing an amino acid substitution that may cause a gain-of-function phenotype.

We further show that both probands are compound heterozygous for the missense or splice site *MYBPC3* mutation and a deep intronic variant in *VCL*. This variant (rs113195070) is predicted to be pathogenic based on three independent computational tools, GWAVA, CADD and Genomiser (**Table 3**). Moreover, it is 3-fold more frequent in our cohort of probands than in normal European populations (**Table 3**), further suggesting a direct contribution to the disease phenotype. The variant, which consists of a single nucleotide substitution located at position 367 from the nearest canonical splice site (c.499+367T>C), can potentially disrupt the binding of transcription factors that have been reported as implicated in pathways related to cardiac regulation, development or pathophysiology such as FOS, JUN and EP300 [55],[56]. By interfering with the binding of transcription regulatory factors, the variant is expected to alter the transcription rate of the *VCL* gene. Consistent with this view, sequence elements located within introns of large human genes have been shown to act as transcriptional enhancers [64], and a recent study reported an *IRF4* gene variant located in intron 4 that strongly affects *IRF4* transcription through disruption of an enhancer element [65].

Analysis of family #6 reveals that the presence of the *VCL* variant or the *MYBPC3* mutation in isolation is not sufficient to cause disease phenotype. Indeed, the two clinically affected individuals in this family are compound heterozygous for the *VCL* variant and the *MYBPC3* splice site mutation (**Fig 3**), suggesting that the combination of the two mutations triggers the disease. A loss-of-function mechanism for the *MYBPC3* mutation could explain why in family #6 only compound heterozygous members manifest the disease, whereas the presence of the heterozygous gain-of-function mutation in family #15 would be sufficient to cause disease. Complex genotypes, including individuals that carry 2 or more variants in the same or different sarcomere-related genes, have been reported in 8% of HCM patients [58], and there is evidence indicating that patients with complex genotype and multiple simultaneous mutations may have more severe or early disease expression [66]. However, complex genotype-phenotype correlations focusing specifically on carriers of splice site mutations remain to be investigated.

We further identified two single nucleotide substitutions in the titin gene (rs142156368 and rs72650063) that are 3 to 6-fold more frequent in our cohort of probands than in normal European populations (**Table 3**). These variants are located in the PEVK domain that plays a role in extensibility of the sarcomere and contractility of the titin protein [67, 68]. Titin is prone to extensive alternative splicing that can change its size and its elastic/stiffness properties; associations have been established between the ratio of expression levels for the main cardiac isoforms (N2BA and N2B) and genetic and non-genetic forms of cardiac diseases [69, 70]. If these variants do interfere with titin splicing, as predicted by the SPIDEX computational model, they are likely to contribute to HCM phenotype, particularly in combination with other HCM-associated alleles. Supporting this view, titin-truncating splicing isoforms, which are encountered in approximately 1% of the general population, are sufficient to induce molecular and physiological effects on the heart [71].

In conclusion, this study provides a framework for scrutinizing variation along the complete sequence of HCM-associated genes and prioritizing candidates for further analysis. Our data suggest that deep intronic variation contributes to HCM phenotype. Translation of genetic information found in an individual to clinical decision taking requires a precise understanding of the molecular mechanisms underlying the disease phenotype. To date, mechanistic and functional studies have been largely restricted to animal models in part due to difficulties in obtaining human tissue from patients. However, the recent emergence of patient-derived induced pluripotent stem cells (iPSCs) that can be differentiated into functional cardiomyocytes recapitulating HCM-specific characteristics [72, 73] holds great promise as an exciting new approach to study how gene mutations relate to clinical outcomes and might be applied to test our hypothesis-generating data.

## Supporting information

S1 Fig Comparison of variant calling strategies. **A)** Variants identified by each individual tool and variants that were independently called by at least two tools (combined) were compared to a standard reference (NA12878, (33)). Concordant or true positive (TP) variants are defined as those present in the reference and identified by the indicated calling tool. Discordant extra or false positive (FP) variants are variants not detected in the reference but identified by the calling tool. Discordant missing or false negative (FN) variants are those present in the reference but undetected by the calling tools. **(B)** Sensitivity was assessed by calculating the ratio between TP/(TP+FN). Precision was assessed by calculating the ratio between TP/(TP+FP).
(TIF)

S2 Fig Characterization of sequence data. **(A)** Box plots show the read-depths across the targeted genes and the average percentage of covered base pairs per gene is depicted in red. **(B)** Box plots show the read-depths in coding (orange) and noncoding (grey) regions. The average percentage of covered base pairs in each region per gene is depicted in red.
(PDF)
