# Supplementary figures and images for: Whole gene sequencing identifies deep-intronic variants with potential functional impact in patients with hypertrophic cardiomyopathy
Source: PLoS One. 2017 Aug 10;12(8):e0182946. doi: 10.1371/journal.pone.0182946 (PMC5552324; doi:10.1371/journal.pone.0182946)

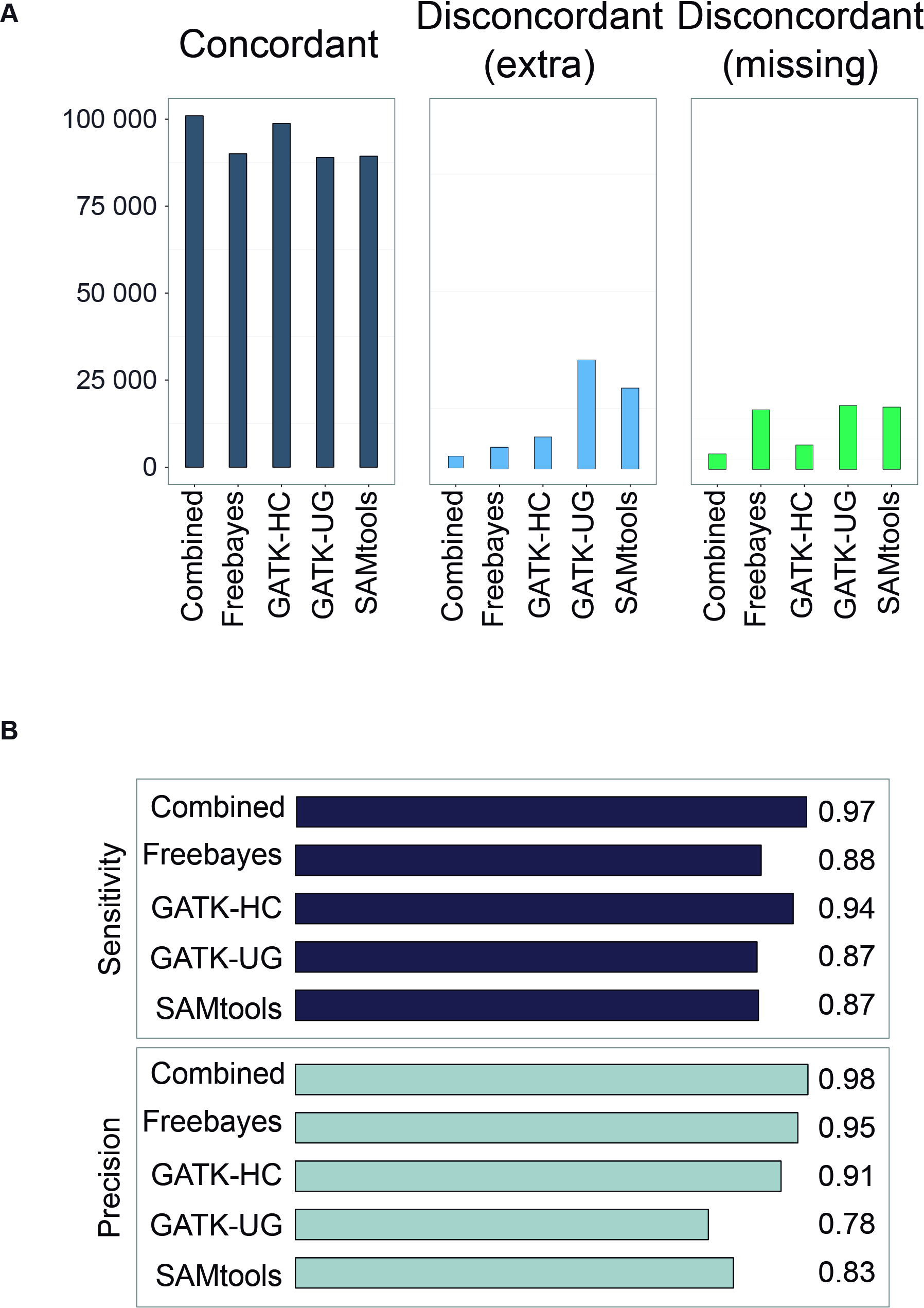

Supplement: S1 Fig — A) Variants identified by each individual tool and variants that were independently called by at least two tools (combined) were compared to a standard reference (NA12878, (33)). Concordant or true positive (TP) variants are defined as those present in the reference and identified by the indicated calling tool. Discordant extra or false positive (FP) variants are variants not detected in the reference but identified by the calling tool. Discordant missing or false negative (FN) variants are those present in the reference but undetected by the calling tools. (B) Sensitivity was assessed by calculating the ratio between TP/(TP+FN). Precision was assessed by calculating the ratio between TP/(TP+FP). (TIF) [file pone.0182946.s001.tif]

A

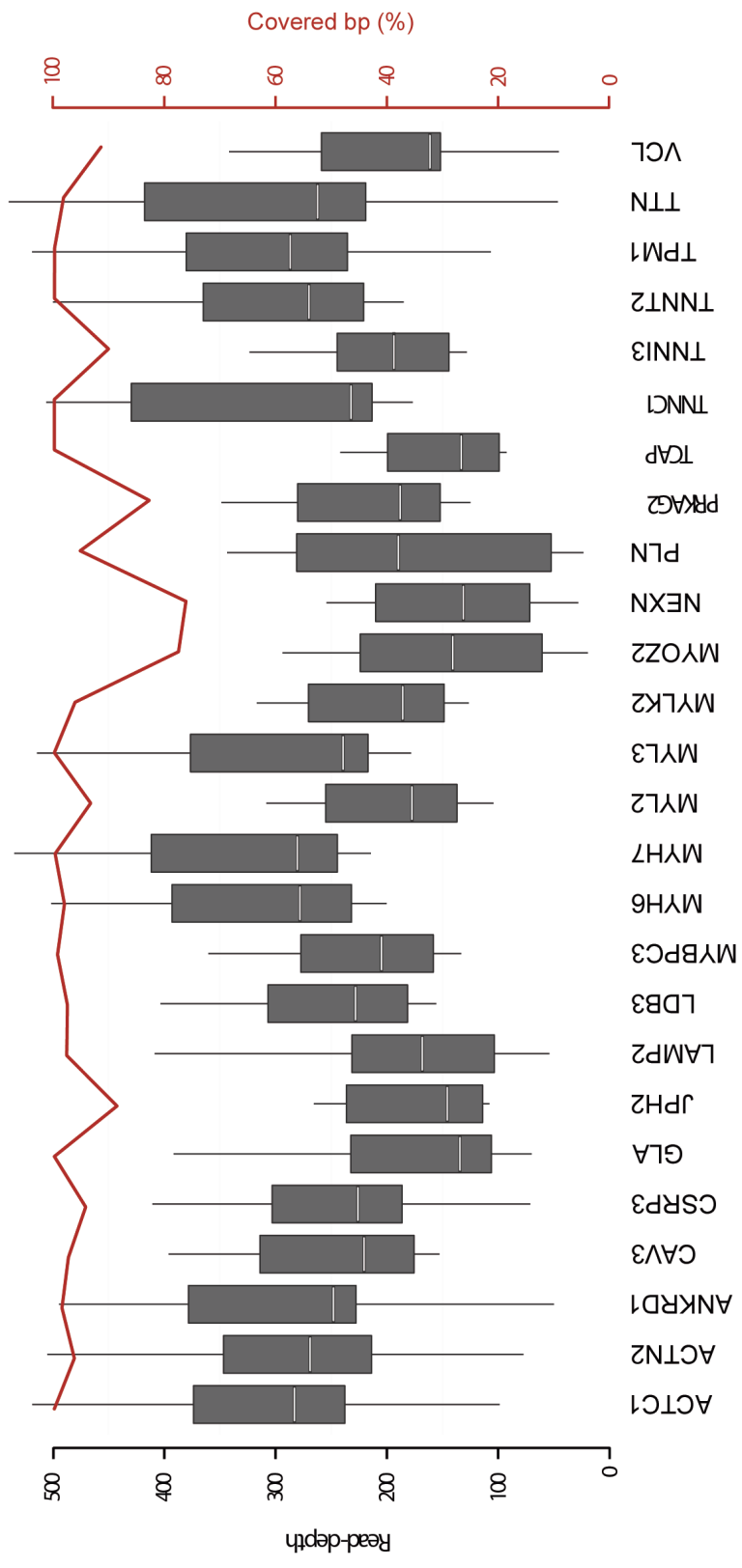

B

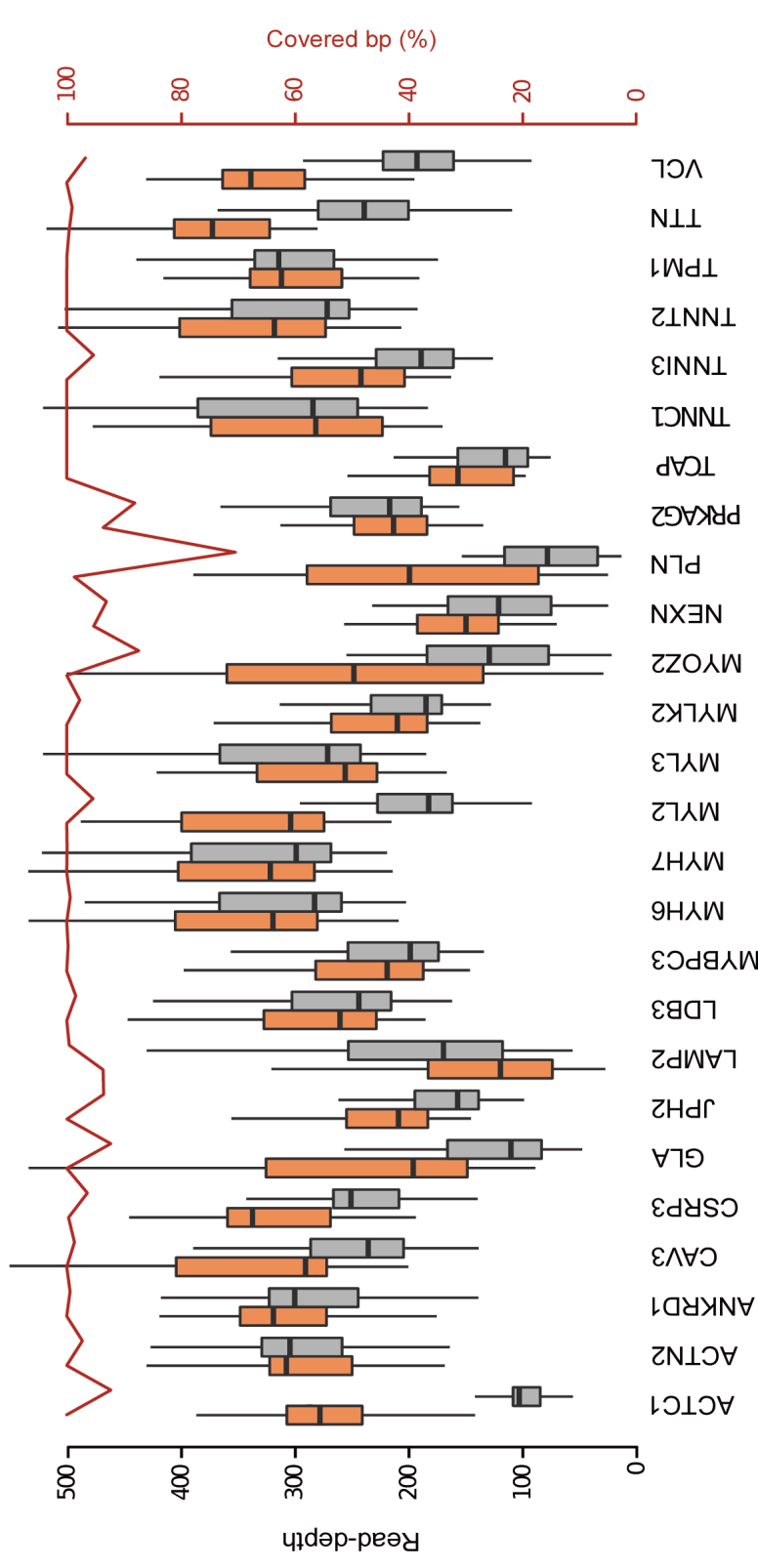

Supplement: S2 Fig — (A) Box plots show the read-depths across the targeted genes and the average percentage of covered base pairs per gene is depicted in red. (B) Box plots show the read-depths in coding (orange) and noncoding (grey) regions. The average percentage of covered base pairs in each region per gene is depicted in red. (PDF) [file pone.0182946.s002.pdf]
